# Oxidative Status of Ultra-Processed Foods in the Western Diet

**DOI:** 10.3390/nu15234873

**Published:** 2023-11-22

**Authors:** Lisaura Maldonado-Pereira, Carlo Barnaba, Ilce Gabriela Medina-Meza

**Affiliations:** 1 Department of Chemical Engineering and Materials Science, Michigan State University, East Lansing, MI 48824, USA; maldon60@msu.edu; 2 Department of Biosystems and Agricultural Engineering, Michigan State University, East Lansing, MI 48824, USA; 3 Department of Pharmaceutical Chemistry, University of Kansas, Lawrence, KS 66047, USA

**Keywords:** dietary oxysterols, phytosterols, ultra-processed foods, cholesterol oxidation products, lipid oxidation

## Abstract

Ultra-processed foods (UPFs) have gained substantial attention in the scientific community due to their surging consumption and potential health repercussions. In addition to their well-established poor nutritional profile, UPFs have been implicated in containing various dietary oxidized sterols (DOxSs). These DOxSs are associated with a spectrum of chronic diseases, including cardiometabolic conditions, cancer, diabetes, Parkinson’s, and Alzheimer’s disease. In this study, we present a comprehensive database documenting the presence of DOxSs and other dietary metabolites in >60 UPFs commonly consumed as part of the Western diet. Significant differences were found in DOxS and phytosterol content between ready-to-eat (RTE) and fast foods (FFs). Biomarker analysis revealed that DOxS accumulation, particularly 25-OH and triol, can potentially discriminate between RTEs and FFs. This work underscores the potential utility of dietary biomarkers in early disease detection and prevention. However, an essential next step is conducting exposure assessments to better comprehend the levels of DOxS exposure and their association with chronic diseases.

## 1. Introduction

Metabolic disorders are linked to poor dietary habits worldwide, where ultra processed foods (UPFs) are emerging as a serious threat to public health [1,2]. UPFs are a risk factor for metabolic disorders, including but not limited to metabolic and cardiovascular disorders, neurodegeneration, and cancer [3,4,5]. Moreover, UPFs have been associated with the development of addictive eating behaviors, driven by the irresistible appeal of high-fat and high-sugar content, leading to enduring alterations in brain function [6,7]. The adverse effect of long-term exposure to UPFs is linked to their high caloric content compared to their lower levels of essential micronutrients such as minerals and vitamins [8]. UPFs have become the most convenient and accessible food option for developed countries, reaching 57% of Americans’ caloric consumption in 2018 [9], where 52.9% of UPFs are mostly consumed away from home by US youth aged 2–19 years [10].

The USDA’s 2020–2025 Dietary Guidelines state that 60% of Americans have one or more preventable chronic diseases related to dietary patterns [11]. In the last decade, the USDA dietary and nutritional guidelines have been highlighting the need for healthy habits; however, these dietary recommendations still need to be met by the population. The most popular UPFs consumed in the USA are fast foods (FFs), including small, large, and non-chain restaurants, and ready-to-eat (RTE) foods, previously processed products packed for sale, which require minimum preparation at home [12,13]. FFs and RTEs are top preferences among children and youth [14,15] because of their flavor, attractive design, and marketing [16,17]. 

While is true that UPFs undergo extensive changes during industrial transformation to preserve microbiological safety and stability [18], including high temperatures, exposure to light, type of cooking, packaging conditions, aging, and storage, a caveat is that UPFs embody an unbalanced ingredient profile. UPFs’ ingredients are mostly constructed from a narrow range of cheap, extracted, refined and fractionated ingredients and detrimental processing that promotes oxidation of sensible compounds such as lipids and cholesterol [19,20], where changes are difficult to discriminate. Thus, the need for a reliable biomarker of processing is needed. 

Dietary oxysterols (DOxSs), oxidized compounds derived from cholesterol in food oxidation processes [21,22], have been proposed as potential markers of thermal food processing [20,23,24]. Our previous research identified 7-ketocholesterol as a marker of spray drying in infant formulations [20]. DOxSs are bioactive lipids known to exert cytotoxic, pro-inflammatory, and pro-apoptotic properties [21,25,26]. The establishment of food processing biomarkers can provide valuable insights beyond mere qualitative classification. Thus, systematic mapping of DOxSs in the food system, particularly in UPFs [9], is imperative for a comprehensive assessment of the impact of processing on the nutritional quality of these foods.

This study aims to profile DOxSs and other bioactive lipids in more than 50 fast food and ready-to-eat meals commonly consumed in the United States, initiating the creation of a comprehensive database accessible to both consumers and the food industry, ultimately enhancing food safety and public health.

## 2. Materials and Methods

### 2.1. UPF Selection, Collection, and Preparation

The UPFs were selected and classified into two main groups: fast foods (FFs) and ready-to-eat, according to our previous study [27]. FFs (n = 23) were collected from retail stores, supermarkets, food chains, restaurants, and takeaway in the Greater Lansing area (Michigan, USA) between February 2018 and October 2019, covering 75% of the national market [13,14,28]. The RTE foods (n = 39) were selected based on the total dietary study 2011–2017 inventory [29,30]. The complete list of the food meals, their respective test codes, and their group is provided in Table 1. Additionally, foods were grouped into categories according to the fat source, as follows: eggs and egg derivatives (E), dairy products (D), meat and poultry (MP), seafood (S), and baby food (BF). Food items that did not fit in any of the previous categories, such as potato products (potato crisps with and without added flavors and French fries from restaurants and takeaway), pasta, salad dressings, and popcorn (sweet or salty) were grouped as other products (O).

FFs were purchased from the selected franchises and brought to the laboratory for immediate analysis. RTE meals were purchased from different local supermarket stores and immediately brought to the laboratory. Storage conditions were followed according to the label instructions (fresh foods were kept in a fridge at 4 °C and frozen meals were kept at −20 °C or the temperature indicated on the label). 

Once the UPF samples arrived at the laboratory, we promptly recorded the following essential information: (1) UPF name; (2) price; (3) place of purchase, date, and time of collection; (4) type of food (ready-to-eat—RTE or fast food—FF); (5) nutritional declaration (including energy, fat, saturated fatty acids, carbohydrates, sugars, fiber, protein, and salt); (6) portion size; (7) list of ingredients; (8) expiration date; and (9) any other pertinent details, such as storage requirements. For certain UPFs that required additional preparation before analysis, we strictly followed the manufacturer’s instructions, which are listed in Table 2. We conducted the analysis using the Sensory Lab kitchen facility at Michigan State University, ensuring accuracy and precision. The experiments were performed with three biological replicates and we stored the samples appropriately, taking into consideration their specific food matrices.

### 2.2. Materials, Chemicals, and Reagents

Methanol was from Sigma-Aldrich. Chloroform was obtained from Omni Solv (Burlington, MA, USA), hexane was purchased from VWR BDH Chemicals (Batavia, IL, USA), 1-butanol and potassium chloride (KCl) from J. T. Baker (Allentown, PA, USA), and diethyl ether was purchased from Fisher Chemical (Pittsburgh, PA, USA). Sodium sulfate anhydrous (Na_2_SO_4_) and sodium chloride [31] were also purchased from VWR BDH Chemicals. Standards of 7α-hydroxycholesterol (7α-OH), 7β-hydroxycholesterol (7β-OH), 5,6α-epoxycholesterol (5,6α-epoxy), 5,6β-epoxycholesterol (5,6β-epoxy), triol, and 7-ketocholesterol (7-keto) were purchased from Steraloids (Newport, RI) and purified using aminopropyl [32] cartridges (500 mg/3 mL) from Phenomenex (Torrance, CA, USA).

### 2.3. Lipid Extraction

A cold lipid extraction was performed according to Folch and coworkers [33], with some modifications depending on the food matrix. Thirty grams of sample were homogenized using an Ultra-Turrax^®^ (Tekmar TP 18/10S1 Cincinnati, OH, USA) for 3 min at 300 rpm and then placed in a 500 mL glass bottle with screw cap with 200 mL of a chloroform:methanol solution (1:1, *v*/*v*). The bottle was kept in an oven at 60 °C for 20 min before adding an additional 100 mL chloroform to have a final 2:1 *v*/*v* chloroform:methanol extraction solution. After 2 min of vortex, the contents of the bottle were filtered. The filtrate was mixed thoroughly with 100 mL of 1 M KCl solution. Samples were left overnight at 4 °C. Then, the lower phase containing lipids was collected and dried at 60 °C with a vacuum evaporator at 25 in Hg. Total fat content was determined gravimetrically and fatty acid profiles were previously reported [27]. In addition, fat content was reported in both units—percentage of total fatty acid weight (% *w*/*w*) per 100 g of fresh sample and weight of total fat per serving size (g per serving size)—for nutritional comparison purposes.

### 2.4. Thiobarbituric Acid Reactive Substances (TBARS)

The method modified by Miller [34] was used to measure lipid oxidation in all UPFs, with some modifications. Briefly, 60 mg of fat was weighed into a 10 mL glass test tube with a screw cap and the following reagents were added: 100 μL BHT solution (0.2 mg/mL in water) and 4.9 mL extracting solution (10% TCA in 0.l M H_2_PO_3_). Sample blanks were analyzed along with each sample. A standard curve of 0–5 mL TEP solution (10 μM) was prepared. Test tubes were incubated overnight in the dark at room temperature. TBARS were expressed as μg malondialdehyde (MDA)/g fat sample.

### 2.5. Total Cholesterol, Tocopherol, and Phytosterol Content

A simultaneous isolation method for cholesterol, tocopherols, and phytosterols developed in our laboratory [20] was used. A total of 200 mg of lipid was mixed with 50 μg of 19-hydroxycholesterol and 140 μg of 5α-cholestane as internal standards for the determination of phytosterols, tocopherols, and total cholesterol. Subsequently, the sample was mixed with 10 mL of 1 N KOH solution in methanol, left under gentle agitation, and covered from the light for at least 15 h. One-tenth of the unsaponifiable matter was subjected to silylation. 

The sample was mixed with 100 μL of pyridine and 100 μL of Sweeley’s reactive mixture (pyridine/hexamethyldisilazane/trimethylchlorosilane, 10:2:1, *v*/*v*/*v*) at 75 °C for 45 min, dried under nitrogen stream, and dissolved in 1 mL of n-hexane. One microliter of the silylated solution was injected into a gas chromatograph (GCMS-QP2010 SE single quadrupole, Shimadzu Corporation (Kyoto, Japan)) fitted with a Zebron™ ZB-5HT (Phenomenex, Torrance, CA, USA) capillary column (30 m × 0.25 mm × 0.25 μm). Oven temperature conditions were set up as follows: 260 to 300 °C at 2.5 °C/min, then from 300 to 320 °C at 8 °C/min, and finally hold at 320 °C for 1 min. The total time of chromatographic separation was 19.5 min. The injector and detector were both set at 320 °C. Helium was used as the carrier gas at a flow rate of 54.0 mL/min, a split ratio at 1:50, and a pressure constant of 134 kPa. 

### 2.6. DOxS Quantification

The other nine-tenths of the sample was used for the quantification of dietary DOxSs. Purification by NH_2_-SPE was performed according to Kilvington et al. [20]. The enriched fraction was recovered and evaporated to dryness under a nitrogen stream. Subsequently, the purified fraction was silylated with Sweeley’s reactive mixture (at 75 °C for 45 min), dried under a nitrogen stream, and dissolved in 1 mL of n-hexane.

DOxS acquisition was performed with one μL of the silylated sample using a GCMS-QP2010 SE single quadrupole (Shimadzu Corporation, Kyoto, Japan). DOxSs were injected into a single quad GCMS-QP2010 SE with the following conditions: from 250 to 280 °C at 2 °C/min, hold at 280 °C for 7 min, and from 280 to 315 °C at 1.5 °C/min. Helium was used as a carrier gas (flow rate of 0.37 mL/min); the split ratio was 1:15 and the pressure 49.2 KPa. The interface temperature for the GC–MS was 320 °C, with the electron multiplier voltage set at 0 kV and the injector at 320 °C. A Zebron ZB-5 fused silica column (30 m × 0.25 mm i.d. × 0.25 um thickness) coated with 5% phenyl polysiloxane (Phenomenex, Torrance, CA, USA) and selected ion monitoring (SIM) mode were used. The total time of chromatographic separation was 40.33 min.

### 2.7. Statistical Analysis

Descriptive statistics were calculated overall, by category, and by food origin. Both mean and confidence intervals (95%) were computed. When comparing RTEs vs. FFs, data did not follow a normal distribution after a density plot analysis and was not homogenous after a residual plot analysis. Therefore, a Mann–Whitney *U*-test was performed at *p* < 0.05 significance level. Statistical differences between food categories were evaluated using the non-parametric Kruskal–Wallis ANOVA by ranks test at *p* < 0.05 significance level. Spearman’s correlation across nutritional and non-nutritional variables was also tested. All the statistical analyses were computed using Rstudio (Version 1.4.1717 © 2009–2021 RStudio, Boston, MA, USA). Principal component analysis was performed in OriginPro (v. 2023, OriginLab, San Francisco, CA, USA) using a correlation matrix on normalized data. Biomarker analysis was performed using a support vector machine (SVM) multivariate analysis using MetaboAnalyst v.5.0. 

## 3. Results

### 3.1. Fat, Sterol Content, and FAME Nutritional Indexes in RTEs and FFs

**Figure 1:**
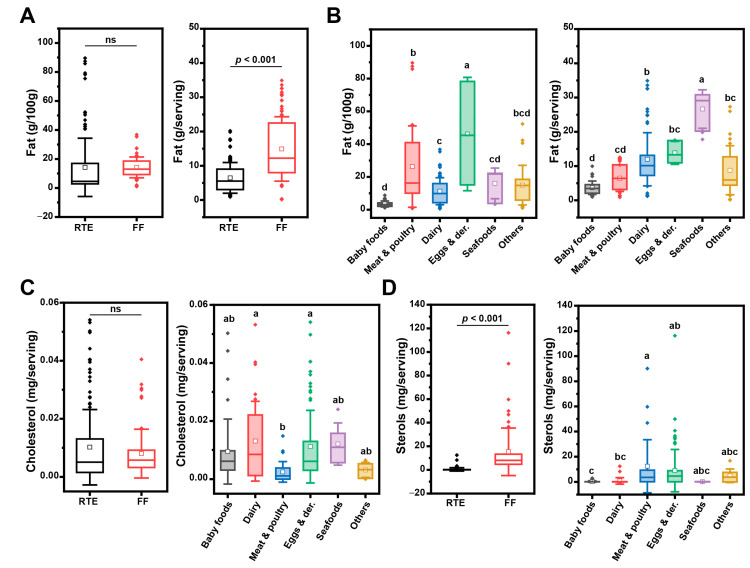
Total fat (**A**,**B**), cholesterol (**C**), and sterol (**D**) content in UPFs by group and category. Box plots represent confidence interval (25–75%) ± 1 SD, line indicates median and white square indicates mean; outliers are also represented as diamonds. When comparing RTEs and FFs a paired *t*-test was used. The grouping letters in the graph represent distinct categories within the variable under analysis, highlighting significant differences between the groups as determined by the ANOVA, followed by post-hoc Tukey test (*p* < 0.05); ns = not statistically different (*p* > 0.05).

We performed quantitative assessment of total fat, cholesterol, and phytosterol content in the selected UPFs (Figure 1). Fat content in UPFs was statistically similar between FFs and RTEs when measured on a weight basis; however, if computed according to the serving size, FFs showed a significantly higher amount of fat (Figure 1A). As expected, fat-enriched food categories were dairy and egg and egg derivatives, whereas seafood categories had a higher fat content when computed on a serving basis (Figure 1B). 

Cholesterol and phytosterols are the sterol-like compounds present in UPFs from animal and vegetable origin, respectively. Our analysis revealed no significant difference in cholesterol content between ready-to-eat (RTE) and fast food (FF) items (Figure 1C). However, when examining specific food categories, we observed only marginal differences in cholesterol content (Figure 1C), with FF displaying a slightly higher sterol content (Figure 1D). Notably, meat and egg products exhibited the most significant variations in sterol content (Figure 1D, right panel). As anticipated, cholesterol levels were notably elevated in dairy and egg products (categories D and E, respectively). In dairy products, dietary cholesterol levels ranged from 80 mg to 110 mg per serving [35]. The lack of vegetable oils and other ingredient sources of phytosterols drove the dairy category to rank fourth in phytosterol content. Interestingly, dairy products also were fourth in DOxS content. The eggs and egg derivatives category had the highest phytosterol content among all categories and was the second highest category in cholesterol content (Figure 1C,D). The UPFs within the eggs category, including mayonnaise (E1-RTE) and macaroni salad (E2-RTE), are not exclusively composed of eggs. They contain a significant amount of soybean oil, which is a main source of phytosterols and places this category in first place compared to the rest of the categories. The position of the eggs category in the cholesterol content assay was as expected, given its well-documented high cholesterol content, ranging from 193 mg to 275 mg per egg [36,37,38]. BFs have high cholesterol content and a discrete amount of phytosterol (Figure 1C) due to their formulation, which includes meat derivatives as main ingredients as well as vegetable oils [20,39,40,41]. Meat and poultry had a lower cholesterol content than the dairy and eggs category (Figure 1C), with a few exceptions. MP6-RTE, MP16-FF, MP17-FF, and BF1-RTE (samples principally made of chicken and beef) contained the highest cholesterol amounts (Table S1, Supplementary Materials). Therefore, the ingredients used during the confection of these meals need to be taken into consideration. The high cholesterol content in the chicken noodle soup (MP6-RTE) can be attributed to the enrichment of the noodles with eggs. Notably, eggs are the primary factor contributing to the elevated cholesterol levels in this meal. Interestingly, in the case of the chicken drumstick (MP16-FF) and chicken wing (MP17-FF) samples, whey (containing 10.3 mg cholesterol per cup) is the ingredient responsible for the cholesterol content. Furthermore, in the case of MP16-FF and MP17-FF, there exists the potential for ingredient accumulation during the frying step, depending on the frequency with which the frying oil in these fast-food preparations is replaced. 

**Figure 2:**
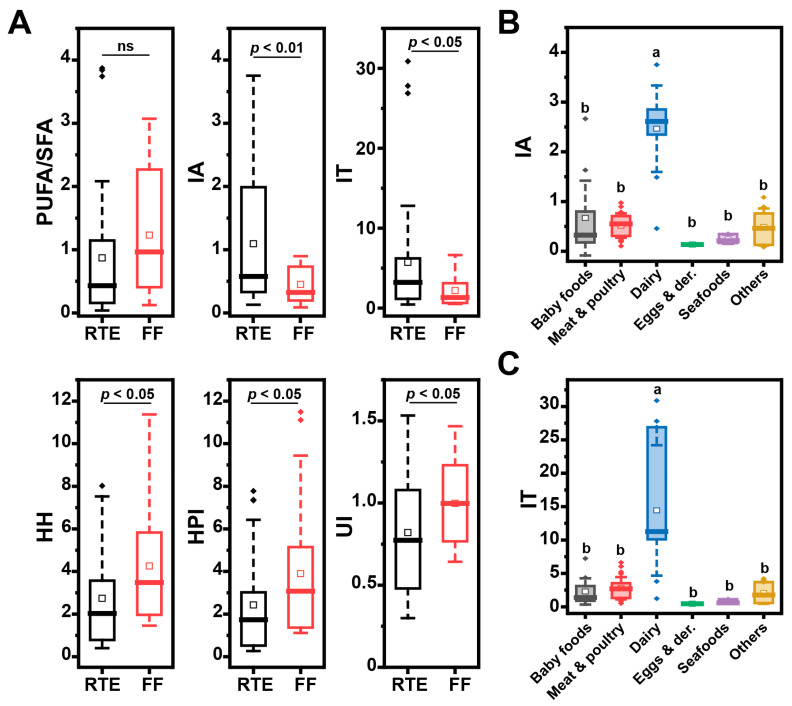
Fatty acid nutritional indexes calculated for UPFs by group and category. (**A**) Nutritional indexes for RTEs and FFs based on their fatty acid composition (IA: index of atherogenicity, IT: index of thrombogenicity, HH: hypocholesterolemic/hypercholesterolemic ratio, HPI: health-promoting index, UI: unsaturated index); (**B**) IA and (**C**) IT indexes for UPFs grouped according to their food category. Box plots represent confidence interval (25–75%) ± 1 SD, line indicates median and white square indicates mean; outliers are also represented as diamonds. The grouping letters in the graph represent distinct categories within the variable under analysis, highlighting significant differences between the groups as determined by the ANOVA, followed by post-hoc Tukey test (*p* < 0.05).

In our previous study, we characterized the fatty acid composition of both RTE and FF classes [27]. Herein, to help further discussion of the oxidative status of UPFs, we computed a series of fatty acid nutritional indexes as reviewed by Chen and Liu [42], based on our previous data (Figure 2A). The PUFA/SFA index is used to determine the impact of fatty acids on cardiovascular health, since a diet rich in PUFAs has been linked to lower serum cholesterol levels [42]. In the present study, no significant difference in PUFA/SFA index was observed between FFs and RTEs. The indexes of atherogenicity (IA) and thrombogenicity (IT) were both proposed in the early 1990s and are linked to specific nutritional attributes of fatty acids. These indexes measure the atherogenic/thrombogenic potential of fatty acids as a ratio between common saturated fatty acids and total unsaturated fatty acids [43]. We calculated these indices for the UPFs profiled in this study (see Section 2). RTEs had a significantly higher IA and IT index value compared to FFs (Figure 2A); dairy foods were the class that most contributed to the high IA and IT values (Figure 1B,C), due to their load of SFAs [27]. Finally, the HH (hypocholesterolemic/hypercholesterolemic ratio), the HPI (health-promoting index) and the UI (unsaturated index) are higher for foods with higher health benefits. In our database, FFs scored higher than RTEs for all three indexes (Figure 2A).

### 3.2. Oxidative Status of the UPFs

**Figure 3:**
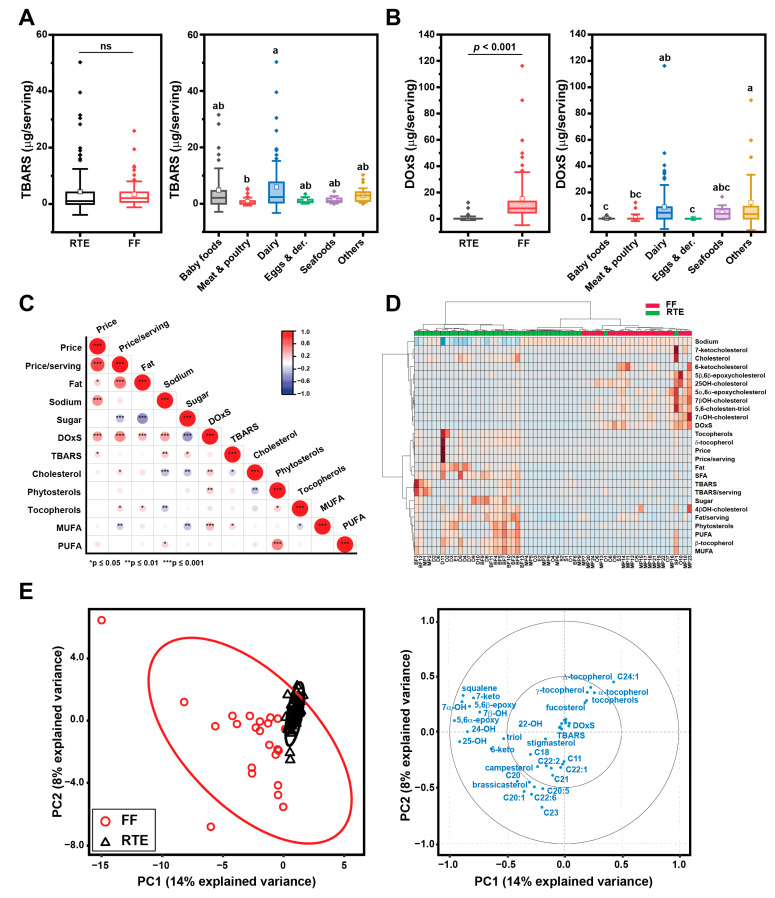
Oxidative status of UPF content in UPFs by group and category. (**A**) TBARS values; (**B**) DOxS amounts. Box plots represent confidence interval (25–75%) ± 1 SD, line indicates median and white square indicates mean; outliers are also represented as diamonds. When comparing RTEs and FFs a paired *t*-test was used. The grouping letters in the graph represent distinct categories within the variable under analysis, highlighting significant differences between the groups as determined by the ANOVA, followed by post-hoc Tukey test (*p* < 0.05). (**C**) Pearson correlation between oxidative status and selected compositional variables; (**D**) heat maps showing clustering of RTEs and FFs according to oxidative markers and selected classes of lipids; warmer colors (i.e., red) indicate higher values for the considered variable. (**E**) principal component analysis of lipids and sterols, showing (left) clustering of RTEs (black solid line) and FFs (red solid line), as well as variable loadings (right).

In this study, we used the TBARS method to detect products of lipid peroxidation, with a focus on malondialdehyde (MDA), which serves as a final product resulting from the peroxidation of fatty acids. MDA concentration values were not significantly different between FFs and RTEs, but significant differences were found between UPF categories (Figure 3A). The highest values for significant amounts of MDA were observed in the meat and poultry category, followed by the baby food and others categories (Table S2, Supplementary Materials). Notably, in several samples, MDA was not detected. 

We then quantified the total amount of DOxSs, which reports cholesterol oxidation. DOxSs are cholesteryl derivatives that accumulate in significant amounts within food matrices because of food manufacturing. Importantly, DOxSs have known biological effects in both in vitro and animal models [44,45]. The FF group showed lower DOxS content (0.15–0.67 µg/serving 95% CI) compared to RTEs (10.5–20.1 µg/serving 95% CI) (Figure 3B). As expected, dairy foods contained higher amounts of DOxSs because they are rich in cholesterol [35,46]. DOxS content was positively correlated with price (both absolute and per serving base), as well as fat and sodium content (*p* < 0.001) (Figure 3C). Conversely, the negative correlation of those macronutrients with cholesterol content reaffirms that DOxS accumulation is indeed driven by food processing. 

### 3.3. DOxS Quantification in UPFs

Twelve DOxSs (7α-OH, 7β-OH, 4β-OH, 5,6α-epoxy, 5,6β-epoxy, triol, 6-keto, 7-keto, 20α-OH, 22-OH, 24-OH, and 25-OH) were identified in FF meals and RTE foods. The DOxS content for individual food samples is reported in the Supplementary Materials (Tables S4 and S5). DOxS contents were significantly higher in the O group, followed by MP and S (Table 1). The most abundant DOxS in all UPFs was 7α-OH, followed by 7β-OH. Notably, 4β-OH was only detected in FF meals. Also, two side-chain COPs (22-OH and 25-OH) were the most abundant specifically in baby food samples. The heat map in Figure 3D highlights compositional differences between FFs and RTEs, which were better resolved using sparse partial least squares discriminant analysis (sPLS-DA) (Figure 3E). For the sPLS-DA we included the FAME data from our previous study published elsewhere [27]. The multivariate analysis showed that RTEs are tightly grouped when considering reduced variables featuring lipid and sterol composition. On the other hand, FFs were broadly distributed across a dimension comprising several DOxSs but also long-chain FAMEs and phytosterols. Taken together, the observed differences in oxidative loads hint that specific compounds may serve as potential biomarkers for processing.

### 3.4. DOxSs as Biomarkers of Food Processing: A Preliminary Assessment

**Figure 4:**
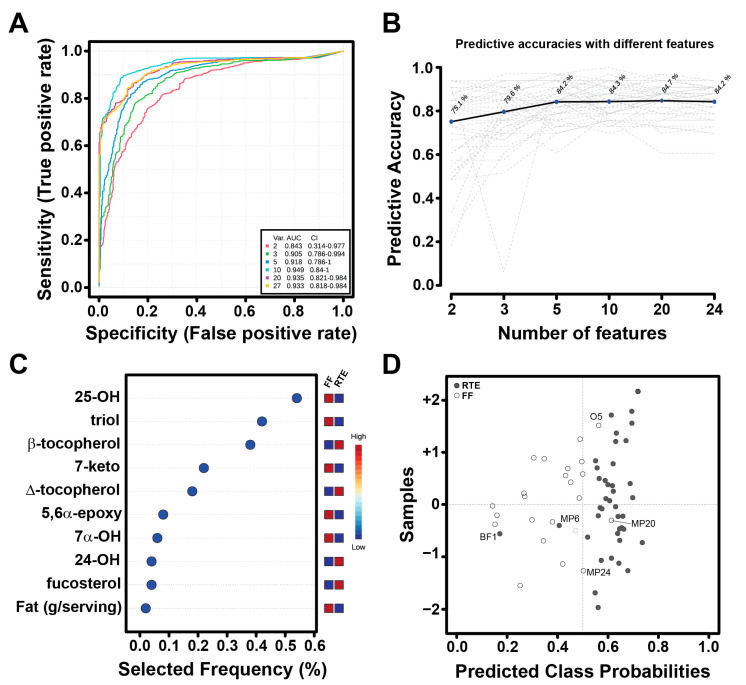
Biomarker analysis for UPFs. (**A**) ROC curves based on cross-validation performance using an SVM classification method and a built-in SVM feature-ranking method. (**B**) Predictive accuracies with increasing number of features. The grey dashed lines represent the performance evaluation performed multiple times through the Monte-Carlo cross validation. (**C**) Top ten significant features with selected frequencies for RTE and FF groups. (**D**) Predicted class probabilities as a result of average cross-validation for each sample using the best classifier (according to the AUC value). Misclassified samples are labeled.

We looked for specific lipid oxidative biomarkers for differentiating food belonging to the FF and RTE categories. To enhance the discriminatory power, we employed a multivariate exploratory ROC analysis to aid in identifying the minimum number of features to incorporate into the model. The support vector machine (SVM) algorithm was then utilized to establish optimal boundaries for classifying distinct groups using a subset of variables (e.g., biomarkers) in multidimensional space. The model predicts high power discrimination between FFs and RTEs even when using only two features (AUC = 0.843), whereas the highest power is achieved with 10 features (AUC = 0.949) (Figure 4A). The accuracy is >75% for all the considered models (Figure 4B). A few DOxSs were selected as top features from the VIP plot (Figure 4C), including 25-OH, triol, and 7-keto, which were previously highlighted in the heat map (Figure 3D). Finally, using the first two features (25-OH and triol), we achieved sufficient discrimination between FFs and RTEs, with only five food items (three meat products, one baby food, and a miscellaneous) assigned to the wrong category.

Overall, our results demonstrate clear differences in the oxidative status of FFs and RTEs, particularly when referring to DOxS content. 

## 4. Discussion

Numerous studies have linked the consumption of UPFs with adverse health outcomes, but there is a lack of quantitative data or biomarkers to assess the level of processing in these food items. Moreover, the variability in manufacturing processes and the sample matrix further complicates the analysis of a food’s composition and its metabolites. In this study, we present an initial evaluation of the oxidative status of UPFs using an onsite database and quantitative lipidomics, focusing on the main lipid oxidative species.

### 4.1. FF and RTE Have Distinct Oxidative Signatures

We previously reported that RTE meals not only have higher fat content compared to FF meals, but also a different FAME composition [27]. These differences are due to the extensive formulation and ingredients used to produce more palatable and tasty meals in addition to the cooking and preservation methods employed during their manufacture. In the present study, we confirmed that formulation also affects the sterol composition of UPFs (Figure 1), and most importantly their oxidative status. Although both groups (FF and RTE) are considered UPFs, there is a clear distinction between these meals due to type of transformation [47], source of lipid and protein (animal-based vs. plant-based), type of preservation technology (e.g., thermal vs. nonthermal, wet versus dry), and addition of additives (e.g., edulcorates, colorants, acidifies). The question this raises is whether the differences in composition and manipulation, from both a manufacturing and preparation point of view, will be reflected in major lipid oxidative markers. For instance, although no difference was observed in secondary oxidative products (measured as MDA), FFs accumulate more DOxSs, which are derived from chemical oxidation of cholesterol (Figure 2A,B). Surprisingly, in several samples the content of MDA was below the limit of detection. This could be due to the absence of second-stage oxidation molecules in those samples or the possibility that the samples had advanced to later stages of the oxidative chain reaction. In these later stages, complex and non-reactive species are formed, rendering them undetectable by our methods. 

### 4.2. DOxSs, UPFs, Nutrition, and Public Health

The recent alterations to dietary guidelines worldwide stem from emerging research findings. Some of these studies propose that cholesterol alone may not be the sole culprit responsible for chronic diseases like CHD. Instead, they indicate that various other factors, such as overall dietary patterns and their impact on the gut microbiota, play a significant role in the development of human pathological conditions. Recent studies have reported association of specific gut metagenomic species with multiple plasma metabolites [48,49,50,51,52,53,54,55]. Characterization of these interactions was stored in an online atlas created by Dekkers and coworkers [56], expecting to help the scientific community in the process of understanding the effects of the gut microbiota on health. It is possible that DOxSs could also be influencing changes in the gut microbiota, which could end up affecting human health [57]. 

### 4.3. Identification of Oxidized Biomarkers

Processing alters both micro- and macronutrients in food and can result in the formation of harmful compounds [21]. Concerning cholesterol oxidation, extensive literature demonstrates that processing-induced oxidation leads to the creation of cholesterol derivatives. Over the past decade, numerous efforts have been undertaken to identify markers of food processing [23,58]. In foods containing animal components, lipids are particularly susceptible to chemical oxidation, prompting a growing interest in discovering lipid-derived processing markers. In this context, the accumulation of dietary oxysterols (DOxSs) has garnered attention due to their direct implication in human pathologies [22,59,60,61]. Our previous work [62] has identified 7-hydroxycholesterol as a potential biomarker for the processing of baby formulas. Other research groups have also suggested that C7 oxidation of cholesterol likely represents a key area for processing-induced cholesterol oxidation, with several C7-derived DOxSs proposed as potential markers of food manufacturing [20]. 

Previous attempts at discovering markers were limited to foods within the same category [20,23]. A more formidable challenge is identifying manufacturing markers that apply across diverse food groups. In this study, we aimed to identify a robust oxidative marker capable of distinguishing between ready-to-eat (RTE) and fast-food (FF) items, utilizing an initial dataset of 63 items. Our approach involved employing multivariate techniques, and the results revealed that a combination of just two dietary oxysterols (DOxSs)—25-OH and triol—achieved an accuracy rate exceeding 75% in distinguishing RTE from FF foods (Figure 4). Further research quantifying DOxSs in a broader dataset of UPFs found in the Western diet is suggested to increase the diet’s variability and evaluate any possible changes in the results obtained from the biomarker analysis. This expanded analysis will provide valuable insights into the specific biomarkers that can best differentiate between different categories of UPFs, contributing to a better understanding of their implications for human health. Consumer awareness of foods and their healthiness is of critical importance [63,64] and varies across cultures. A pilot study performed in Europe found that Italian and Dutch consumers have a weaker negative opinion toward UPFs compared to Brazilian consumers [63]. However, healthiness perception of UPFs seems to be linked to heuristic assessment [64]. For instance, the National Institutes of Health (NIH) recognizes the need for more data to provide insight into personalized foods for precision nutrition [65]. A dietary assessment will enable the development of an exposure assessment that would become the starting point of a greatly needed risk assessment to elucidate the association between these food meals and disease risk [66]. 

## 5. Conclusions

Our study revealed significant differences in DOxS content between RTE and FF meals. Similarly, the levels of phytosterols exhibited a significant difference between RTE and FF meals. These findings underscore the substantial influence of the parameters and conditions used in the manufacturing processes of these two groups of UPFs. Furthermore, the composition of the food matrix played a pivotal role in determining the presence of these bioactive lipids, as observed when comparing all six food categories. Overall, not all UPFs are deemed to be “unhealthy”, a lot of these compounds, such as phytosterols and tocopherols, are needed to avoid oxidative species that promote development or worsening of human illnesses. Unfortunately, the presence of DOxSs in UPFs increases the risk of cardiometabolic disorders (CMD), which are one of the most challenging global health issues of the 21st century, recognized by the World Health Organization (WHO) [67] and the American Society of Endocrinology [68]. Oxidative compounds like these cholesterol oxidation products [62] need to be avoided for the sake of society’s health.

Therefore, we believe that the use of DOxSs as biomarkers could potentially help in the future to identify the presence of different chronic diseases in their early stages, and even prevent their development. Nevertheless, an exposure assessment is critical to understand the exposure level of these toxic compounds—DOxSs—and their relationship with CMD and other chronic diseases. 

No nutritional database of UPFs has been fully developed yet. Maldonado and coworkers already analyzed the nutritional aspects of the same 63 UPFs evaluated in this study [27]. Our results aim to expand that database, which will serve as our contribution to the scientific community and general society in the ongoing quest of elucidating the relationship between DOxSs and human health. The creation of a DOxS database of UPFs in WD developed in this study is just the first step to assess and determine the potential health risk caused by the consumption of these UPFs.

## Figures and Tables

**Table 1:** Ultra-processed food meal IDs used in this study, divided by group and category.

Category	Sample ID	Ultra-Processed Foods (UPFs)	Group
Dairy	D1—RTE	American cheese—Happy Farms	Ready to Eat (RTE)
	D2—RTE	Cheddar cheese—Happy Farms	
	D3—RTE	Margarine (regular, not low-fat, salted)—Countryside Creamery	
	D5—RTE	Cream (half and half)—Meijer	
	D6—RTE	Swiss cheese—Kroger	
	D7—RTE	Cream cheese—Happy Farms	
	D8—RTE	Ice cream (regular, not low-fat, vanilla)—Purple Cow	
	D9—RTE	Yogurt (low-fat, fruit flavored)—Yoplait	
	D10—RTE	Chocolate milk—Nesquick	
	D11—RTE	Infant formula—Little Journey (with iron milk-based powder)	
	* D4—RTE	Butter—Prairie Farms	
Meat and Poultry	MP1—RTE	Bologna—Eckrich	
	MP2—RTE	Salami—Oscar Mayer	
	MP3—RTE	Bean soup w/bacon/pork (canned, prepared w/water)—Campbell’s	
	MP4—RTE	Chili con carne w/beans (canned)—Campbell’s	
	MP5—RTE	Lasagna w/meat (frozen, heated)—Michael Angelo’s	
	MP6—RTE	Chicken noodle soup—Kroger	
	MP7—RTE	Beef and vegetable soup—Kroger	
	MP8—RTE	Mini ravioli—Chef Boyardee	
	MP9—RTE	Spaghetti—Chef Boyardee	
Seafood	S1—RTE	Clam chowder (New England, canned, prep w/whole milk)—Kroger	
Eggs and derivatives	E1—RTE	Mayonnaise (regular, bottled)—Hellmann’s	
	E2—RTE	Macaroni salad (from grocery/deli)—Meijer	
Baby food	BF1—RTE	Baby food—beef and broth/gravy—Beech Nut	
	BF2—RTE	Baby food—chicken and broth/gravy—Gerber	
	BF3—RTE	Baby food—vegetables and beef—Gerber	
	BF4—RTE	Baby food—vegetables and chicken—Gerber	
	BF5—RTE	Baby food—chicken noodle dinner—Gerber	
	BF6—RTE	Baby food—macaroni, tomato and cheese—Gerber	
	BF7—RTE	Baby food—turkey and rice—Gerber	
	BF8—RTE	Baby food—turkey and broth/gravy—Beech Nut	
	BF9—RTE	Baby food—fruit yogurt—Gerber	
	BF10—RTE	Baby food—chicken with rice—Gerber	
	BF11—RTE	Baby food—vegetables and turkey—Gerber	
	BF12—RTE	Baby food—macaroni and cheese with vegetables—Gerber	
	BF13—RTE	Pasta pick-ups (cheese ravioli)—Gerber	
Other	O1—RTE	Popcorn w/butter (microwave)—Kroger	
	O2—RTE	Salad dressing (creamy/buttermilk type, regular)—Aldi’s Tuscan Garden	
	O3—RTE	Macaroni and cheese (boiled)—Kraft	
	O4—RTE	Macaroni and cheese (microwaved)—Kraft	
Meat and poultry	MP10—FF	Hamburger on bun—McDonald’s	Fast food (FF)
	MP11—FF	Chicken nuggets—McDonald’s	
	MP12—FF	Cheeseburger on bun—McDonald’s	
	MP13—FF	Steak tacos w/beans, lettuce, rice and cheese—Chipotle	
	MP14—FF	Cheese and chicken quesadilla—Chipotle	
	MP15—FF	Chicken burrito w/lettuce, cheese, and pico—Chipotle	
	MP16—FF	Chicken drumstick—KCF	
	MP17—FF	Chicken wing—KFC	
	MP18—FF	Beef w/vegetables—Panda Express	
	MP19—FF	Chicken w/vegetables—Panda Express	
	MP20—FF	Chicken filet—(broiled sandwich)—Chick Fil’A	
	MP21—FF	Roast beef, ham and provolone—Jimmy Johns	
	MP22—FF	Sliced turkey and bacon—Jimmy Johns	
	MP23—FF	Supreme pizza—Marco’s Pizza	
	MP24—FF	Pepperoni pizza, hand tossed—Domino’s	
Seafood	S2—FF	Fish sandwich on bun—McDonald’s	
	S3—FF	Fried shrimp—Panda Express	
Others	O5—FF	French fries—McDonald’s	
	O6—FF	McDonald’s biscuit—Big Breakfast	
	O7—FF	McDonald’s hotcakes—Big Breakfast	
	O8—FF	Biscuit—KFC	
	O9—FF	French fries—KFC	
	O10—FF	Mashed potato—KFC	

* The only processed (category 3 NOVA) food in this study.

**Table 2:** Additional cooking preparations according to the label instructions.

Food Group	Sample	Sample Collection	Cooking Conditions	Lipid Extraction
Seafood	S1-RTE		Microwave: High heat for 2 min and then for 30 s at a time until cooked, stirring each time, about 3 min	Entire product used
	S2-FF	McDonald’s	-	Entire product except for the bun
Meat and Poultry	MP3-RTE		Microwave: Covered on high heat for 2 ½ to 3 min	Entire product used
	MP4-RTE			
	MP5-RTE		Oven bake: 25–35 min at 400 °F	
	MP6-RTE		Microwave: Covered, on high heat for 4 to 5 min or until hot	
	MP7-RTE			
	MP8-RTE		Microwave: 1 min 30 s or until warm on high heat	
	MP9-RTE			
	MP10-FF	McDonald’s	-	Entire product except for the bun
	MP11-FF	McDonald’s	-	Entire product used
	MP12-FF	McDonald’s	-	Entire product except for the bun
	MP13-FF	Chipotle	-	Entire product except for the tortilla
	MP14-FF	Chipotle	-	Entire product used
	MP15-FF	Chipotle	-	Entire product except for the tortilla
	MP16-FF	KFC	-	Entire product except for the bones
	MP17-FF	KFC	-	Entire product except for bones
	MP20-FF	Chick Fil’A	-	Entire product except for the bun
	MP21-FF	Jimmy John’s	-	Entire product except for the bread
	MP22-FF	Jimmy John’s	-	
Others	O1-RTE		Microwave: 2–2 ½ min on high heat	
	O2-RTE		-	
	O3-RTE		Boil pan: Stir for 7 to 8 min in boiling water	
	O4-RTE		Microwave: Uncovered, on high heat 8 to 10 min or until water is absorbed, stirring every 3 min	Entire product used

- Not applicable.

## Data Availability

Data available upon request to the corresponding authors.

## Supplementary Materials

The following supporting information can be downloaded at: https://www.mdpi.com/article/10.3390/nu15234873/s1, Table S1: Cholesterol content in UPFs by group and food category; Table S2: MDA concentration in RTE items and FF meals; Table S3: Phytosterols content in UPFs; Table S4: DOxS content in RTE group; Table S5: DOxS content in FF group.
