# Supplementary material for: Oxidative Status of Ultra-Processed Foods in the Western Diet
Source: Nutrients. 2023 Nov 22;15(23):4873. doi: 10.3390/nu15234873 (PMC10708126; doi:10.3390/nu15234873)
Supplement: Supplementary file 1 [file nutrients-15-04873-s001.zip › nutrients-2711793-supplementary.pdf]

Table S1: Cholesterol content in UPFs by group and food category. (Total values per category are expressed in mean and range).

READY-TO-EAT				
Food Category	***Sample ID***	***Cholesterol (mg/100 g fat) ± STD***	***Cholesterol Reference (mg/100 g fat)***	***Serving Size***
Dairy	D1-RTE	20.30 ± 15.85	333.33	19.00 g
	D2-RTE	196.33 ± 29.43	333.33	21.00 g
	D3-RTE	ND	N/A	1 tbsp
	*D4-RTE	305.90 ± 41.16	272.73	14.00 g
	D5-RTE	165.09 ± 30.33	285.71	30.00 mL
	D6-RTE	287.08 ± 20.03	300.00	28 .00g
	D7-RTE	238.86 ± 133.50	285.71	28.00 g
	D8-RTE	243.02 ± 40.22	357.14	0.50 cup (65.00 g)
	D9-RTE	368.25 ± 72.77	0.00	1 container (99.00 g)
	D10-RTE	282.18 ± 52.16	500.00	414.03 g
	D11-RTE	16.25 ± 3.59	NR	1 scoop (9 g)
	Total	**20.3 (0 – 368.25)**		
Baby food	BF1-RTE	1,247.23 ± 227.45	1,000.00	71.00 g
	BF2-RTE	673.84 ± 98.50	833.33	4.00 oz (113.40 g)
	BF3-RTE	58.17 ± 8.50	250.00	4.00 oz (113.40 g)
	BF4-RTE	319.43 ± 83.74	500.00	4.00 oz (113.40 g)
	BF5-RTE	348.65 ± 82.26	400.00	4.00 oz (113.40 g)
	BF6-RTE	123.24 ± 29.61	7.81	128.00 g
	BF7-RTE	263.77 ± 76.69	400.00	4.00 oz (113.40 g)
	BF8-RTE	683.55 ± 310.63	875.00	71.00 g
	BF9-RTE	162.73 ± 95.67	333.33	4.00 oz (113.40 g)
	BF10-RTE	251.02 ± 57.95	333.33	4.00 oz (113.40 g)

	BF11-RTE	313.34 ± 27.54	285.71	4.00 oz (113.40 g)
	BF12-RTE	172.87 ± 17.84	333.33	4.00 oz (113.40 g)
	BF13-RTE	327.40 ± 87.35	500.00	85.00 g
	Total	**380.40 (50.17 – 1,247.23)**		
Meat & Poultry	MP1-RTE	224.75 ± 15.73	333.33	38.00 g
	MP2-RTE	648.56 ± 125.37	250.00	32.00 g
	MP3-RTE	327.69 ± 28.27	200.00	240.00 mL
	MP4-RTE	157.80 ± 55.79	500.00	1 can (236.59 g)
	MP5-RTE	396.25 ± 46.38	500.00	11.00 oz (227.00 g)
	MP6-RTE	2,023.90 ± 209.36	750.00	0.5 cup (113.40 g)
	MP7-RTE	352.50 ± 166.24	583.33	0.5 cup (240.00 g)
	MP8-RTE	62.55 ± 8.88	142.86	1 cup (249.00 g)
	MP9-TE	199.16 ± 96.55	181.82	1 cup (257.00 g)
	Total	**488.13 (62.55 – 2023.90)**		
Seafood	S1-RTE	43.89 ± 1.81	50.00	18.80 oz (532.97 g)
	Total	**43.89**		
Eggs and derivatives	E1-RTE	98.44 ± 16.35	90.91	1 tbsp
	E2-RTE	274.11 ± 40.94	218.75	0.50 cup (115.00 g)
	Total	**186.28 (98.44 – 274.11)**		
Others	O1-RTE	ND	NR	2 tbsp (30.00 g)
	O2-RTE	51.55 ± 11.01	66.67	2 tbsp (30.00g)
	O3-RTE	97.51 ± 32.67	333.33	2.5 oz (70.90 g)
	O4-RTE	37.95 ± 8.46	285.71	1 package (58.00 g)
	Total	**62.34 (37.95 – 97.51)**		
FAST FOOD				
Food Category	***Sample ID***	***Cholesterol (mg/100 g fat) ±STD***	***Cholesterol Reference (mg/100 g fat)***	***Serving Size***

Meat & Poultry	MP10-FF	370.80 ± 55.74	267.22	95.00 g
	MP11-FF	229.99 ± 53.08	222.05	4 pieces (64.00 g)
	MP12-FF	362.03 ± 8.90	296.86	119.00 g
	MP13-FF	446.38 ± 73.73	464.29	1 taco (102.00 g)
	MP14-FF	299.73 ± 46.37	230.77	1 quesadilla (170.00 g)
	MP15-FF	504.08 ± 7.82	245.31	1 burrito (140.00 g)
	MP16-FF	742.86 ± 62.13	820.56	1 piece (75.00 g)
	MP17-FF	728.22 ± 42.86	600.00	1 piece (60.00 g)
	MP18-FF	333.59 ± 105.66	171.43	5.40 oz (153. 09 g)
	MP19-FF	310.55 ± 147.36	1,423.08	5.70 oz (161.69 g)
	MP20-FF	526.02 ± 61.70	312.92	1 sandwich (187.00 g)
	MP21-FF	450.17 ± 13.91	272.73	1 sandwich (71.28 g)
	MP22-FF	335.74 ± 109.38	240.74	1 sandwich (94.61 g)
	MP23-FF	280.77 ± 38.10	NA	1 slice (123.00 g)
	MP24-FF	266.45 ± 30.50	200.00	1 slice (79.00 g)
	Total	**387.21 (8.00 – 742.86)**		
Seafood	S2-FF	216.98 ± 26.53	204.39	1 sandwich (131.00 g)
	S3-FF	405.14 ± 37.49	555.56	5.00 oz (141.75 g)
	Total	**311.06 (216.98 – 405.14)**		
Others	O5-FF	ND	0.00	1 medium serving (117.00 g)
	O6-FF	ND	222.99	1 biscuit (76.00 g)
	O7-FF	268.72 ± 43.02	0.00	3 hotcakes (149.00 g)
	O8-FF	16.42 ± 8.31	5.87	1 biscuit (49.00 g)
	O9-FF	ND	0.00	1 order (34.99 g)
	O10-FF	90.13 ± 21.47	0.00	1 order (16.85 g)

	Total	**62.55 (16.42 – 268.72)**	

ND = not detected

N/A = not apply

NR = not reported

Table S2: MDA concentration in RTE items and FF meals.

**Overall UPFs**		
Food Category	***µg MDA/g fat ±STD***	***Kruskal-Wallis Test***
Dairy	0.785 ± 1.42	*p=0.0114*
Meat & Poultry	5.97 ± 9.23	
Baby Food	4.86 ± 7.70	
Eggs and Derivatives	1.20 ± 1.16	
Seafood	1.40 ± 1.34	
Others	2.95 ± 2.54	

**READY-TO-EAT**		
Food Category	***Food Item***	***µg MDA/g fat ±STD***
Dairy	D1-RTE	3.58 ± 1.28
	D2-RTE	0.36 ± 0.23
	D3-RTE	-
	D4-RTE	-
	D5-RTE	1.03 ± 0.052
	D6-RTE	0.19 ± 0.017
	D7-RTE	0.37 ± 0.27
	D8-RTE	-
	D9-RTE	-
	D10-RTE	3.22 ± 2.18
	D11-RTE	-
	Average ±STD	***0.785 ±1.39***
Meat & Poultry	MP1-RTE	29.66 ± 8.43
	MP2-RTE	12.67 ± 3.42
	MP3-RTE	7.59 ± 0.23

	MP4-RTE	7.73 ± 5.87
	MP5-RTE	2.12 ± 1.48
	MP6-RTE	-
	MP7-RTE	-
	MP8-RTE	6.76 ± 4.83
	MP9-RTE	23.83 ± 18.69
	Average ±STD	***9.68 ±12.48***
Baby food	BF1-RTE	-
	BF2-RTE	-
	BF3-RTE	19.42 ± 7.41
	BF4-RTE	-
	BF5-RTE	3.30 ± 0.87
	BF6-RTE	7.95 ± 5.47
	BF7-RTE	2.47 ± 0.42
	BF8-RTE	2.81 ± 1.43
	BF9-RTE	1.48 ± 0.24
	BF10-RTE	9.36 ± 0.51
	BF11-RTE	2.51 ± 1.09
	BF12-RTE	17.87 ± 11.01
	BF13-RTE	-
	Average ±STD	***4.86 ±7.61***
Eggs and Derivatives	E1-RTE	0.56 ± 0.042
	E2-RTE	1.85 ± 1.19
	Average ±STD	***1.20 ±1.06***
Seafood	S1-RTE	1.26 ± 0.66
	Average ±STD	***0.84 ±0.80***
Others	O1-RTE	1.88 ± 1.46
	O2-RTE	3.03 ± 0.89

	O3-RTE	3.83 ± 0.60
	O4-RTE	1.24 ± 0.23
	Average ±STD	***2.49 ± 1.36***
**Overall RTE Average ± STD**		***4.30 ± 8.14***

**FAST FOOD**		
Food Category	***Food Item***	***µg MDA/g fat ±STD***
Meat & Poultry	MP10-FF	4.15 ± 1.14
	MP11-FF	1.35 ± 0.79
	MP12-FF	-
	MP13-FF	3.43 ± 1.70
	MP14-FF	-
	MP15-FF	3.64 ± 2.24
	MP16-FF	-
	MP17-FF	-
	MP18-FF	17.01 ± 6.32
	MP19-FF	2.13 ± 0.47
	MP20-FF	1.20 ± 0.71
	MP21-FF	4.36 ± 1.24
	MP22-FF	3.41 ± 1.95
	MP23-FF	1.53 ± 0.69
	MP24-FF	11.20 ± 7.77
	**Average ± STD**	**3.75 ± 5.25**
Seafood	S2-FF	2.66 ± 1.25
	S3-FF	0.69 ± 0.38
	Average ±STD	***1.67 ±1.35***
Others	O5-FF	7.27 ± 2.61
	O6-FF	1.84 ± 1.64
	O7-FF	2.11 ± 0.90

	O8-FF	-
	O9-FF	3.22 ± 1.86
	O10-FF	6.20 ± 2.40
	**Average ± STD**	**3.25 ± 2.99**
**Overall FF Average ± STD**		**3.44 ± 4.60**

- Means No detected

Table S3: Phytosterol content in UPFs. (Total values of phytosterols are expressed as mean and range.)

Ready to Eat						
Food Category	***Food Item***	***Brassicasterol***	***Campesterol***	***Stigmasterol***	***β-Sitosterol***	***Fucosterol***
		(mg/100 g fat) ± STD				
Dairy	D1-RTE	ND	26.01 ± 9.31	20.30 ± 6.01	86.64 ± 12.43	ND
	D2-RTE	ND	ND	ND	ND	ND
	D3-RTE	ND	33.31 ± 14.70	43.37 ± 12.51	136.05 ± 5.72	ND
	D4-RTE	ND	ND	ND	ND	ND
	D5-RTE	ND	ND	ND	ND	ND
	D6-RTE	ND	ND	ND	ND	ND
	D7-RTE	ND	ND	ND	ND	ND
	D8-RTE	ND	ND	ND	ND	ND
	D9-RTE	ND	ND	ND	ND	ND
	D10-RTE	ND	ND	ND	ND	ND
	D11-RTE	ND	18.19 ± 4.86	11.64 ± 5.32	95.02 ± 24.75	ND
	**Total**	**-**	**26.01 (18.19 – 33.31)**	**25.10 (11.64 – 43.37)**	**105.90 (86.64 – 136.05)**	**-**
Meat & Poultry	MP1-RTE	ND	ND	ND	ND	ND
	MP2-RTE	ND	ND	ND	tr	ND
	MP3-RTE	ND	68.45 ± 13.99	259.84 ± 40.55	626.48 ± 57.01	58.18 ± 26.67
	MP4-RTE	ND	ND	31.84 ± 9.45	102.34 ± 29.25	ND
	MP5-RTE	ND	14.60 ± 5.20	ND	ND	0.033 ± 0.026
	MP6-RTE	ND	ND	ND	ND	ND
	MP7-RTE	ND	ND	ND	ND	ND
	MP8-RTE	ND	36.47 ± 24.74	tr	83.75 ± 43.50	ND
	MP9-RTE	ND	ND	ND	ND	ND
	**Total**	**-**	**39.84 (14.60 – 68.45)**	**145.84 (31.84 – 259.84)**	**270.86 (83.75 – 626.48)**	**29.11 (0.03 – 58.18)**
Seafood	S1-RTE	ND	33.99 ± 26.75	tr	51.87 ± 37.21	ND

	Total	-	33.99	tr	51.87	-

Table S3: Phytosterol content in UPFs (cnt'd). (Total values of phytosterols are expressed as mean and range.)

Ready to Eat						
Food Category	***Food Item***	***Brassicasterol***	***Campesterol***	***Stigmasterol***	***β-Sitosterol***	***Fucosterol***
		(mg/100 g fat) ±STD				
Eggs & egg's derivatives	E1-RTE	ND	61.64 ± 24.67	42.06 ± 13.92	176.90 ± 18.66	ND
	E2-RTE	ND	53.46 ± 6.89	37.17 ± 9.46	162.59 ± 24.23	ND
	**Total**	**-**	**57.55 (53.46 – 61.64)**	**39.62 (37.17 – 42.06)**	**169.75 (162.59 – 176.90)**	**-**
Baby foods	BF1-RTE	ND	ND	ND	ND	ND
	BF2-RTE	ND	ND	ND	ND	ND
	BF3-RTE	ND	10.48 ± 5.14	6.52 ± 1.87	47.10 ± 13.21	ND
	BF4-RTE	ND	182.76 ± 53.20	75.16 ± 41.86	498.98 ± 129.71	ND
	BF5-RTE	ND	136.48 ± 47.29	33.77 ± 7.19	391.77 ± 93.78	ND
	BF6-RTE	ND	21.98 ± 6.84	ND	100.31 ± 19.76	ND
	BF7-RTE	ND	190.88 ± 21.15	ND	421.59 ± 137.27	ND
	BF8-RTE	ND	ND	ND	ND	ND
	BF9-RTE	ND	ND	ND	ND	ND
	BF10-RTE	ND	48.06 ± 9.69	25.41 ± 14.33	160.14 ± 26.56	ND
	BF11-RTE	19.72 ± 3.21	ND	48.31 ± 2.23	435.62 ± 41.06	ND
	BF12-RTE	29.60 ± 3.30	99.33 ± 24.73	ND	236.18 ± 18.19	ND
	BF13-RTE	ND	44.86 ± 3.55	ND	ND	0.046 ± 0.014
	**Total**	**24.66 (19.72 – 29.60)**	**91.85 (10.48 – 190.88)**	**37.83 (6.52 – 75.16)**	**286.46 (47.10 – 498.98)**	**0.046**
Others	O1-RTE	ND	34.54 ± 5.32	ND	ND	ND
	O2-RTE	ND	50.87 ± 14.28	38.09 ± 10.27	148.17 ± 8.02	ND
	O3-RTE	ND	38.65 ± 19.12	ND	ND	5.45 ± 1.58
	O4-RTE	ND	26.01 ± 9.31	16.34 ± 9.22	124.15 ± 12.47	39.85 ± 17.26

	Total	-	**37.52 (26.01 – 50.87)**	**27.22 (16.34 – 38.09)**	**136.16 (124.15 – 148.17)**	**22.65 (5.45 – 39.85)**

Table S3: Phytosterol content in UPFs (cnt'd). (Total values of phytosterols are expressed as mean and range.)

Fast Food						
Food Category	***Food Item***	***Brassicasterol***	***Campesterol***	***Stigmasterol***	***β-Sitosterol***	***Fucosterol***
		(mg/100 g fat) ±STD				
Meat & Poultry	MP10-FF	ND	ND	ND	ND	ND
	MP11-FF	ND	122.85 ± 18.26	ND	296.67 ± 38.94	ND
	MP12-FF	ND	ND	ND	ND	ND
	MP13-FF	ND	66.46 ± 14.08	26.36 ± 10.01	176.24 ± 26.14	ND
	MP14-FF	ND	81.36 ± 13.67	209.33 ± 40.88	ND	ND
	MP15-FF	ND	ND	53.03 ± 7.46	ND	ND
	MP16-FF	ND	86.20 ± 33.23	ND	165.55 ± 63.66	ND
	MP17-FF	27.56 ± 4.66	101.79 ± 8.40	ND	173.08 ± 17.49	ND
	MP18-FF	ND	68.98 ± 23.39	48.25 ± 13.69	343.35 ± 137.26	ND
	MP19-FF	ND	4.77 ± 3.44	ND	33.40 ± 17.52	ND
	MP20-FF	ND	22.44 ± 7.49	8.93 ± 7.17	95.24 ± 9.40	ND
	MP21-FF	ND	ND	ND	39.39 ± 6.79	ND
	MP22-FF	ND	20.69 ± 10.72	ND	34.28 ± 4.46	ND
	MP23-FF	ND	83.95 ± 7.74	168.10 ± 24.33	ND	ND
	MP24-FF	ND	23.57 ± 7.87	ND	68.94 ± 6.79	ND
	**Total**	**27.56**	**62.10 (4.77 – 122.85)**	**85.67 (8.93 – 209.33)**	**142.61 (33.40 – 343.35)**	**-**
Seafood	S2-FF	ND	60.10 ± 22.86	ND	199.39 ± 26.35	ND
	S3-FF	ND	25.42 ± 4.88	19.73 ± 13.05	36.94 ± 4.58	ND
	**Total**	**-**	**42.76 (25.42 – 60.10)**	**19.73**	**118.17 (36.94 – 199.39)**	**-**
Others	O5-FF	ND	192.00 ± 21.55	ND	331.87 ± 34.11	ND
	O6-FF	ND	ND	36.45 ± 17.21	ND	ND

	O7-FF	ND	40.86 ± 5.16	224.87 ± 30.79	ND	ND
	O8-FF	ND	35.93 ± 23.68	ND	74.36 ± 8.20	ND
	O9-FF	44.19 ± 17.67	184.08 ± 25.17	ND	333.41 ± 45.98	ND
	O10-FF	ND	60.98 ± 12.72	ND	102.64 ± 19.30	tr
	**Total**	**44.19**	**102.77 (35.93 – 192.00)**	**130.66 (36.45 – 224.87)**	**210.57 (74.36 – 333.41)**	**tr**

ND = not detected, tr = traces

Table S4: DOxS content in RTE group, expressed in mg per 100g of fat.

	Dairy												
	7α-OH	*7β-OH*	*4β-OH*	*5,6α-Epoxy*	*5,6β-Epoxy*	*7-Keto*	*Triol*	*6-Keto*	*20α-OH*	*22-OH*	*24-OH*	*25-OH*	*Total COPs*
	(mg/100 g fat) ±STD												
D1-RTE	0.15 ± 0.049	0.14 ± 0.080	-	-	-	-	0.0	-	-	-	-	1.05 ± 1.21	63.59 ± 25.32
D2-RTE	0.084 ± 0.034	0.086 ± 0.026	-	-	-	0.044 ± 0.029	0.051 ± 0.0050	-	-	-	-	-	0.55 ± 0.13
**D3-RTE**	-	-	-	-	-	-	-	-	-	-	-	0.053 ± 0.020	0.14 ± 0.048
**D4-RTE**	0.64 ± 0.29	0.51 ± 0.21	-	0.044 ± 0.025	0.050 ± 0.019	0.37 ± 0.13	0.18 ± 0.12	0.046 ± 0.036	-	-	-	-	2.00 ± 0.92
**D5-RTE**	0.19 ± 0.0061	0.12 ± 0.029	-	-	-	0.11 ± 0.016	-	-	-	-	-	-	1.29 ± 0.39
**D6-RTE**	0.32 ± 0.10	0.27 ± 0.036	-	-	-	0.11 ± 0.025	0.051 ± 0.0056	-	-	-	-	-	1.72 ± 0.26
**D7-RTE**	0.51 ± 0.26	0.39 ± 0.20	-	0.023 ± 0.0046	0.058 ± 0.011	0.20 ± 0.094	0.14 ± 0.032	0.046 ± 0.020	-	-	-	-	1.41 ± 0.52
**D8-RTE**	0.039 ± 0.019	0.043 ± 0.018	-	-	-	-	-	-	-	-	-	-	0.21 ± 0.081
**D9-RTE**	0.36 ± 0.056	0.35 ± 0.072	-	-	-	0.16 ± 0.020	0.10 ± 0.013	0.065 ± 0.038	-	-	-	-	4.06 ± 0.98
D10-RTE	2.75 ± 1.88	4.72 ± 3.26	-	0.20 ± 0.16	-	1.40 ± 0.92	2.49 ± 1.84	0.19 ± 0.038	-	0.15 ± 0.068	-	0.43 ± 0.26	16.09 ± 8.01

D11-RTE	0.075 ± 0.0094	0.09 ± 0.033	-	-	-	-	-	-	0.055 ± 0.032	-	-	-	0.90 ± 0.061

Meat & Poultry													
	7α-OH	*7β-OH*	*4β-OH*	*5,6α-Epoxy*	*5,6β-Epoxy*	*7-Keto*	*Triol*	*6-Keto*	*20α-OH*	*22-OH*	*24-OH*	*25-OH*	*Total COPs*
	(mg/100 g fat) ±STD												
MP1-RTE	5.95 ± 5.43	4.83 ± 4.30	-	0.10 ± 0.064	0.11	1.93 ± 1.57	0.15 ± 0.076	0.065 ± 0.029	-	0.068 ± 0.042	-	0.39 ± 0.32	15.27 ± 12.80
MP2-RTE	4.20 ± 1.20	3.76 ± 1.06	-	0.12 ± 0.026	0.13 ± 0.040	1.14 ± 0.41	0.058 ± 0.0071	0.052 ± 0.020	-	-	-	0.36 ± 0.12	10.25 ± 2.80
**MP3-RTE**	0.081 ± 0.019	0.060 ± 0.027	-	-	-	0.015 ± 0.0084	-	-	-	-	-	-	0.25 ± 0.027
**MP4-RTE**	0.96 ± 0.14	1.13 ± 0.19	-	-	-	0.29 ± 0.021	0.22 ± 0.072	-	-	0.10 ± 0.034	-	0.14 ± 0.095	6.99 ± 1.85
**MP5-RTE**	0.42 ± 0.11	0.32 ± 0.024	-	-	-	0.16 ± 0.099	0.082 ± 0.012	0.079 ± 0.020	-	0.17 ± 0.0081	0.050 ± 0.031	0.11 ± 0.021	2.17 ± 0.22
**MP6-RTE**	15.85 ± 3.91	16.07 ± 4.20	-	0.26 ± 0.12	0.41 ± 0.13	3.75 ± 0.68	2.33 ± 0.37	-	-	-	0.40 ± 0.084	-	43.12 ± 8.98
**MP7-RTE**	0.14 ± 0.029	0.15 ± 0.060	-	-	-	0.066 ± 0.044	-	-	-	-	-	-	0.36 ± 0.13
**MP8-RTE**	0.54 ± 0.062	0.47 ± 0.066	-	-	0.13 ± 0.022	0.12 ± 0.039	0.053 ± 0.020	-	-	0.66 ± 0.043	-	0.19 ± 0.0078	3.09 ± 0.16
**MP9-RTE**	0.27 ±	0.20 ± 0.070	-	-	-	0.085 ± 0.025	-	-	-	-	-	-	1.36 ± 0.38

	0.067												

Seafood

	7α-OH	*7β-OH*	*4β-OH*	*5,6α-Epoxy*	*5,6β-Epoxy*	*7-Keto*	*Triol*	*6-Keto*	*20α-OH*	*22-OH*	*24-OH*	*25-OH*	*Total COPs*
	(mg/100 g fat) ±STD												
**S1-RTE**	0.052 ± 0.020	0.060 ± 0.013	-	-	-	-	-	-	-	0.090 ± 0.013	-	-	0.25 ± 0.026

Eggs & egg's derivatives

	7α-OH	*7β-OH*	*4β-OH*	*5,6α-Epoxy*	*5,6β-Epoxy*	*7-Keto*	*Triol*	*6-Keto*	*20α-OH*	*22-OH*	*24-OH*	*25-OH*	*Total COPs*
	(mg/100 g fat) ±STD												
**E1-RTE**	0.11 ± 0.051	0.13 ± 0.024	-	-	-	0.062 ± 0.021	-	-	0.025 ± 0.017	0.069 ± 0.077	-	0.22 ± 0.036	0.91 ± 0.15
**E2-RTE**	0.47 ± 0.27	0.32 ± 0.19	-	-	-	0.18 ± 0.088	-	-	-	-	-	-	1.35 ± 0.60

Baby Food

	7α-OH	*7β-OH*	*4β-OH*	*5,6α-Epoxy*	*5,6β-Epoxy*	*7-Keto*	*Triol*	*6-Keto*	*20α-OH*	*22-OH*	*24-OH*	*25-OH*	*Total COPs*
	(mg/100 g fat) ±STD												
BF1-RTE	8.97 ± 5.42	24.61 ± 15.21	-	0.69 ± 0.36	1.39 ± 0.81	16.69 ± 5.45	2.63 ± 0.46	-	-	-	-	3.60 ± 1.22	62.55 ± 25.55
BF2-RTE	2.92 ± 0.73	2.27 ± 0.64	-	0.044 ± 0.012	0.033 ± 0.0062	0.84 ± 0.34	0.22 ± 0.031	0.11 ± 0.064	-	-	-	0.24 ± 0.055	8.48 ± 2.46
**BF3-RTE**	0.10 ± 0.031	0.065 ± 0.037	-	-	-	-	-	-	0.65 ± 0.17	0.33 ± 0.11	0.18 ± 0.47	-	2.65 ± 0.46
**BF4-RTE**	1.80 ± 0.22	1.61 ± 0.24	-	-	-	0.57 ± 0.065	0.27 ± 0.086	-	-	0.20 ± 0.044	-	0.16 ± 0.056	7.32 ± 1.08

**BF5-RTE**	2.10 ± 0.50	1.94 ± 0.37	-	0.052 ± 0.021	0.045 ± 0.027	0.59 ± 0.18	0.31 ± 0.091	-	-	0.10 ± 0.034	-	0.27 ± 0.16	6.44 ± 1.23
**BF6-RTE**	3.73 ± 1.98	0.66 ± 0.12	-	-	-	0.67 ± 0.42	0.20 ± 0.034	-	-	0.077 ± 0.031	-	-	8.04 ± 3.16
**BF7-RTE**	2.12 ± 0.16	2.38 ± 0.15	-	-	-	0.48 ± 0.15	0.39 ± 0.056	-	-	0.17 ± 0.069	-	-	6.13 ± 0.30
**BF8-RTE**	5.85 ± 1.82	4.91 ± 1.12	-	0.030 ± 0.019	0.073 ± 0.039	1.68 ± 0.61	0.27 ± 0.10	0.12 ± 0.018	-	-	-	0.67 ± 0.16	14.02 ± 3.97
**BF9-RTE**	0.68 ± 0.12	0.59 ± 0.17	-	-	-	0.24 ± 0.075	0.21 ± 0.050	-	-	-	-	-	8.14 ± 5.70
BF10-RTE	2.08 ± 0.63	1.62 ± 0.46	-	0.027 ± 0.016	0.042 ± 0.032	0.65 ± 0.087	0.35 ± 0.041	-	-	0.067 ± 0.015	-	0.27 ± 0.047	6.36 ± 1.36
BF11-RTE	0.66 ± 0.092	0.58 ± 0.020	-	-	1.05 ± 0.12	-	0.12 ± 0.036	-	-	2.48 ± 1.45	-	-	5.28 ± 1.49
BF12-RTE	0.41 ± 0.10	0.40 ± 0.078	-	-	-	0.18 ± 0.013	0.10 ± 0.044	-	-	0.18 ± 0.11	-	-	2.05 ± 0.31
BF13-RTE	4.22 ± 3.77	1.27 ± 0.89	-	0.062 ± 0.023	0.10 ± 0.025	1.31 ± 0.83	0.15 ± 0.03	-	-	0.12 ± 0.017	0.14 ± 0.029	0.47 ± 0.32	11.58 ± 7.07
						Others							
	7α-OH	***7β-OH***	***4β-OH***	***5,6α-Epoxy***	***5,6β-Epoxy***	***7-Keto***	***Triol***	***6-Keto***	***20α-OH***	***22-OH***	***24-OH***	***25-OH***	***Total COPs***
						(mg/100 g fat) ±STD							
O1-RTE	0.12 ± 0.018	0.094 ± 0.023	-	-	-	-	0.053 ± 0.014	0.023 ± 0.011	-	0.099 ± 0.027	-	-	1.56 ± 0.28
O2-RTE	0.11 ± 0.037	0.089 ± 0.015	-	-	-	0.035 ± 0.0079	0.060 ± 0.016	-	0.031 ± 0.020	-	-	-	0.61 ± 0.12
O3-RTE	0.57 ± 0.11	0.60 ± 0.14	-	0.10 ± 0.030	0.055 ± 0.043	0.20 ± 0.060	0.13 ± 0.055	-	-	0.083 ± 0.0022	-	-	5.60 ± 1.28
O4-RTE	1.19 ± 0.78	1.22 ± 0.79	-	0.066 ± 0.028	0.054 ± 0.053	0.30 ± 0.095	0.17 ± 0.063	0.078 ± 0.020	-	0.051 ± 0.027	-	0.15 ± 0.10	9.58 ± 2.73

Table S5: DOxS content in Fast Food group, expressed in mg per 100 g of fat.

	Meat & Poultry												
	7α-OH	***7β-OH***	***4β-OH***	***5,6α-E***	***5,6β-E***	***7-Keto***	***Triol***	***6-Keto***	***20α-OH***	***22-OH***	***24-OH***	***25-OH***	***Total COPs***
	(mg/100 g fat) ±STD												
**MP10**	3.06 ± 1.37	2.28 ± 1.21	-	0.051 ± 0.0052	0.068 ± 0.024	0.92 ± 0.48	0.12 ± 0.057	-	-	-	0.14 ± 0.094	0.21 ± 0.12	8.64 ± 3.04
**MP11**	0.31 ± 0.031	0.29 ± 0.048	-	-	-	0.12 ± 0.052	0.033 ± 0.015	0.045 ± 0.034	0.11 ± 0.060	0.21 ± 0.17	-	0.17 ± 0.013	1.91 ± 0.084
**MP12**	1.00 ± 0.29	0.72 ± 0.28	-	-	-	0.35 ± 0.083	0.030 ± 0.020	0.050 ± 0.0052	-	-	0.10 ± 0.024	0.11 ± 0.044	3.18 ± 0.65
**MP13**	0.26 ± 0.023	0.28 ± 0.041	-	-	-	0.077 ± 0.16	0.024 ± 0.012	-	-	-	-	0.12 ± 0.036	1.67 ± 0.20
**MP14**	0.75 ± 0.044	0.55 ± 0.086	-	0.056 ± 0.038	0.13 ± 0.041	0.54 ± 0.21	0.21 ± 0.037	0.070 ± 0.021	0.13 ± 0.036	0.20 ± 0.030	-	0.22 ± 0.038	14.64 ± 11.61
**MP15**	1.89 ± 0.66	0.78 ± 0.10	0.49 ± 0.030	0.061 ± 0.013	0.11 ± 0.016	0.86 ± 0.33	0.12 ± 0.040	-	-	-	-	-	5.02 ± 1.08
**MP16**	0.69 ± 0.20	0.75 ± 0.22	-	0.056 ± 0.037	0.044 ± 0.023	0.20 ± 0.033	0.053 ± 0.017	-	0.050 ± 0.017	-	-	-	3.90 ± 1.00
**MP17**	0.19 ± 0.44	0.19 ± 0.037	-	-	-	0.63 ± 0.018	0.031 ± 0.018	-	-	-	-	-	1.55 ± 0.38
**MP18**	3.04 ± 0.32	0.99 ± 0.42	-	0.023 ± 0.011	0.029 ± 0.0099	0.57 ± 0.089	0.067 ± 0.023	0.039 ± 0.014	-	-	-	-	5.72 ± 0.99
**MP19**	0.48 ± 0.044	0.34 ± 0.036	-	0.030 ± 0.020	0.028 ± 0.0057	0.20 ± 0.040	0.11 ± 0.026	0.021 ± 0.0069	-	0.10 ± 0.032	-	0.049 ± 0.029	1.75 ± 0.077
**MP20**	0.18 ± 0.060	0.16 ± 0.040	-	-	-	0.057 ± 0.035	0.037 ± 0.031	-	-	-	-	0.090 ± 0.055	0.91 ± 0.11
**MP21**	4.01 ± 0.081	3.50 ± 0.14	-	0.060 ± 0.019	0.12 ± 0.041	1.42 ± 0.24	0.11 ± 0.038	0.069 ± 0.052	0.019 ± 0.0073	0.019 ± 0.016	0.18 ± 0.029	0.12 ± 0.0099	10.40 ± 0.71

**MP22**	1.81 ± 0.23	2.61 ± 0.29	-	-	-	0.71 ± 0.034	0.071 ± 0.019	-	-	-	-	-	5.40 ± 0.56
**MP23**	16.80 ± 9.61	10.23 ± 4.14	0.41 0.22	0.21 ± 0.078	0.52 ± 0.28	-	0.60 ± 0.32	0.22 ± 0.081	0.16 ± 0.045	0.43 ± 0.11	-	1.29 ± 0.64	39.13 ± 18.94
**MP24**	0.14 ± 0.036	0.18 ± 0.035	-	-	-	-	0.036 ± 0.012	-	-	-	-	0.15 ± 0.064	2.53 ± 0.33
Seafood													
	7α-OH	***7β-OH***	***4β-OH***	***5,6α-Epoxy***	***5,6β-Epoxy***	***7-Keto***	***Triol***	***6-Keto***	***20α-OH***	***22-OH***	***24-OH***	***25-OH***	***Total COPs***
(mg/100 g fat) ±STD													
**S2**	0.33 ± 0.075	0.23 ± 0.029	-	-	-	0.15 ± 0.012	0.092 ± 0.029	-	-	-	-	0.16 ± 0.079	2.66 ± 0.44
**S3**	1.37 ± 0.83	1.27 ± 0.72	-	0.055 ± 0.040	0.18 ± 0.037	0.43 ± 0.25	0.12 ± 0.069	0.081 ± 0.034	0.12 ± 0.065	0.20 ± 0.025	-	0.17 ± 0.042	7.27 ± 2.85
Others													
	7α-OH	***7β-OH***	***4β-OH***	***5,6α-Epoxy***	***5,6β-Epoxy***	***7-Keto***	***Triol***	***6-Keto***	***20α-OH***	***22-OH***	***24-OH***	***25-OH***	***Total COPs***
(mg/100 g fat) ±STD													
**O5**	0.096 ± 0.036	0.093 ± 0.033	0.031 ± 0.0090	0.021 ± 0.0041	-	0.084 ± 0.028	-	-	-	-	0.15 ± 0.039	-	3.16 ± 1.11
**O6**	0.52 ± 0.31	0.30 ± 0.13	-	-	-	0.26 ± 0.18	0.10 ± 0.017	-	0.16 ± 0.10	0.18 ± 0.017	-	tr	1.97 ± 0.61
**O7**	0.81 ± 0.038	0.60 ± 0.044	-	0.068 ± 0.036	0.080 ± 0.018	0.22 ± 0.021	0.075 ± 0.015	-	0.11 ± 0.042	-	-	-	2.40 ± 0.14
**O8**	0.11 ± 0.073	0.096 ± 0.045	-	-	-	-	0.021 ± 0.013	-	-	0.024 ± 0.019	-	0.16 ± 0.033	3.14 ± 1.56
**O9**	0.099 ± 0.037	0.082 ± 0.033	-	-	-	0.056 ± 0.027	0.033 ± 0.0065	-	0.052 ± 0.013	-	-	0.19 ± 0.049	9.62 ± 2.24
**O10**	0.42 ± 0.052	0.37 ± 0.060	-	-	0.10 ± 0.022	-	0.041 ± 0.017	-	-	0.52 ± 0.030	-	0.15 ± 0.011	2.33 ± 0.21

- NO Detected, FF with a blue background, RTE with green background.
